# PREVALENCE OF TUBERCULOSIS, DRUG-RESISTANT TUBERCULOSIS AND HIV/TB CO-INFECTION IN ENUGU, NIGERIA

**DOI:** 10.21010/ajid.v15i2.5

**Published:** 2021-03-18

**Authors:** Kennethe Okonkeo Ugwu, Martin Chinonye Agbo, Ifeoma Maureen Ezeonu

**Affiliations:** † Department of Microbiology, University of Nigeria, Nsukka, Nigeria; †† Department of Pharmaceutical Microbiology and Biotechnology, University of Nigeria, Nsukka

**Keywords:** *Mycobacterium tuberculosis*, Nigeria, MDRTB, Enugu Nigeria, HIV, Xpert^®^ MTB/Rif

## Abstract

**Background:**

Tuberculosis (TB) remains a global public health problem, with developing countries bearing the highest burden. Nigeria is first in Africa and sixth in the world among the countries with the highest TB burden, but is among the 10 countries accounting for over 70% of the global gap in TB case detection and notification. Enugu State, Nigeria reportedly has a notification gap of almost 14,000 TB cases; a situation which must be addressed.

**Materials and Methods:**

A total number of 868 individuals accessing DOTS services in designated centres within the six Local Government Areas (LGAs) of Enugu North geographical zone, was recruited into the study. The participants were screened for HIV seropositivity by standard protocols, while screening for TB and drug-resistant TB were conducted by a combination of Zhiel Neelsen staining and Nucleic Acid Amplification Test (Xpert^®^ MTB/Rif).

**Results:**

Of the 868 subjects that participated in the study, 176 (20.3%) were HIV seropositive. The highest prevalence (26.7%) of HIV was recorded in Udenu LGA, while the least (13.1%) was recorded in Nsukka LGA. Overall TB prevalence was found to be 22.1% and 21.3% by sputum-smear and NAAT, respectively. Uzo Uwani LGA recorded the highest prevalence of both TB (33.3%) and TB/HIV co-infection (16.7%), but the lowest prevalence of resistant TB. Nsukka LGA had the highest prevalence of resistant TB.

**Conclusion:**

Enugu North geographical zone, Nigeria, has a high prevalence of both HIV and TB, including resistant TB and there is need to increase monitoring of individuals resident in this region.

## Introduction

Tuberculosis (TB) remains a global public health problem and one of the top ten leading causes of death, worldwide, with developing countries bearing the highest burden (WHO, 2020). In 2018, Nigeria was listed as first in Africa and sixth among the 30 countries of the world with the highest TB burden (WHO, 2020). Unfortunately, the problem of TB in Nigeria has been complicated by the emergence and spread of drug resistant TB and a high burden of HIV/AIDS (NTBLCP, 2017; WHO, 2018). The problem of TB is worsened when there is also a high burden of HIV infections, as people with HIV are more likely to develop active TB. According to WHO reports, an estimated 63,000 Nigerians living with HIV/AIDS develop TB, while about 39,000 die from the disease, each year (WHO, 2018).

To further compound the problem, Nigeria is ranked among the 10 countries that accounted for 77% of the global gap in TB case detection and notification in 2016. It is reported that Nigeria contributes about 8% of the 4.3 million TB cases missed globally.

Consequently, in 2019, WHO initiated ‘community-informant’ methods to find missing TB cases in Nigerian communities (WHO, 2019). The initiative covered 12 high burden states in Nigeria, but Enugu State was not part of the study. However, according to NTBLCP (2019), Enugu State has a notification gap of almost 14,000 TB cases.

This study was conducted in Enugu North geographical zone. The zone comprises six Local Government Areas (LGAs), namely, Nsukka, Igbo-Etiti, Uzo-Uwani, Igbo Eze North, Udenu and Igbo Eze South, and had a total ward population of about 1,466,996 at last count (NPC, 2012). The zone is characterized by low socio-economic indicators and a majority of the residents engage in low-income generating activities, such as petty trading and peasant farming. In addition, one of the commercial nerve-centres of the zone, Obollo Afor, in Udenu LGA, is also a stopover for commercial drivers and people on transit to and from the northern parts of the country, making the area a hub for commercial sex activity, with attendant problems, such as HIV transmission. The study was, therefore, conducted to determine the prevalence of TB and MDRTB in the six different LGAs of Enugu north geographical zone, with a view to fill some of the gap currently existing in TB detection and reporting in Nigeria.

## Materials and Methods

### Study area

**Figure 1:**
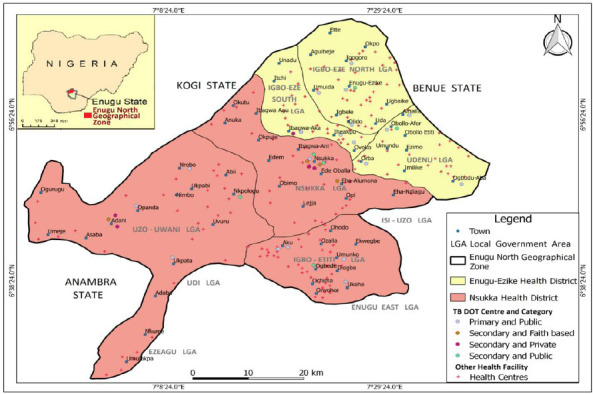
Map of Enugu North Geographical Zone Showing the DOTS Centres.

The study was conducted in Enugu north geographical zone, south eastern Nigeria, which currently constitute Enugu north senatorial zone in the state geopolitical arrangement and is comprised of six local government areas (LGAs). The zone has a total ward population of about 1,466,996 (NPC, 2012). The zone is characterized by low socio-economic indicator, with a majority of the residents engaging in low-income generating activities, such as small trading and peasant farming. The geographical zone has about 35 DOTS centres spread across the six LGAs. Sputum samples were collected from five comprehensive HIV/AIDS treatment sites (2 public health facilities and 3 faith-based organizations) and the other DOTS/ TB Microscopy centres in the zone (Figure 1).

### Study population

The study group comprised 868 individuals of different age groups (from age 5 and above) accessing DOTS services in the designated centres across the 6 LGAs in the zone; with productive cough or presumptive diagnosis of tuberculosis, who volunteered to participate in the study. The survey population for NAAT and drug resistant TB comprised 207 stratified randomly selected individuals with productive cough, accessing clinical services in the 35 DOTS centres. The DOTs centres provided the opportunity to collect sputum samples from individuals with high suspicion index for tuberculosis, those newly diagnosed and those on treatment but on follow up visit to the facilities. Individuals less than 5 years and/or ≥ 5 years who clinically/symptomatically screened positive to tuberculosis but did not have productive cough were excluded from the study.

### Ethical consideration

The study was carried out in accordance with the tenets of the declaration of Helsinki. The purpose of this study was explained to the subjects in English and Igbo languages. Code numbers rather than names were used to identify them to ensure confidentiality. An Informed Consent Form was developed, explained and administered to clients/clients’ care givers (for children from 5 and above). Only consenting clients were enrolled in the study. Participation in the study was completely voluntary and at no cost to the consenting clients.

Clients were also informed that quality of care in the health facility would not in any way, be affected by their decision to participate or not. Approval was obtained from the authorities of district hospitals and the respective DOTS/TB microscopy centres in the zone. Ethical clearance was obtained from the Health Research and Ethics Committee of Enugu State Ministry of Health, Enugu (Ref. No.: MH/MSD/EC/0183).

### Diagnosis of HIV infection

HIV testing was performed on all participants according to the national guidelines for HIV testing, using Determine, Unigold and Stat Pak rapid diagnostic test kits, according to the manufacturers’ instructions. The Determine kit was used as the first line strip for HIV testing, according to the national guidelines. Positive results were retested with the Unigold kit. However, in the event of a discordant result, Stat Pak was used. The final result of the algorithm for a particular sample was scored positive if at least two of the three rapid screening HIV assays were reactive. HIV counseling was offered to all subjects with unknown HIV status and those who previously tested negative.

### Collection and processing of sputum samples

The subjects were advised to rinse their mouth at least twice with water before producing the specimen in order to remove food and reduce contaminating bacteria load from their mouths. They were instructed to take two breaths, and then cough deeply and expectorate the sputum into provided 50 ml screw-capped translucent bottles, holding the sample container close to their mouth. Three sputum specimens (spot, early morning, spot) were collected from each participant under close supervision. The samples were then processed for the standard acid-fast direct smear microscopy using Ziehl-Neelsen staining and one of the spot samples processed for Rifampicin resistance (MDRTB) assay by Nucleic Acid Amplification Test (NAAT) at Enugu Ezike District Hospital TB laboratory, according to the National guidelines. Stained smears were examined for acid-fast bacilli (AFB) under the microscope using oil immersion (100X) objectives.

### Confirmation of TB and drug-resistant TB

Confirmation of the presence of *M. tuberculosis* and resistance (MDRTB) were assayed by Nucleic Acid Amplification Test (NAAT), using the GeneXpert/MTB/RIF Kit, in a semi-nested rt-PCR reaction. The tests were run following the manufacturer’s instructions.

## Results

Local Government and gender distribution of participants

A total number of 868 individuals was enrolled in the study comprising 420 (48.4%) males and 448 (51.6%) females. Of the 868 subjects from the six LGAs in the zone, Igbo Eze North had the highest number of participants, with 425 (48.9%) and Igbo Etiti had the least number of participants, with 29 (3.3%), as shown in Table 1.

**Table 1:** Local Government and Gender Distribution of Participants (N = 868)

LGA	Number of DOTS Centres	Number of Males (%)	Number of Females (%)	Total (%)
Igbo Etiti	5	18 (2.1)	11(1.3)	29 (3.3)
Igbo Eze North	5	187(21.5)	238 (27.4)	425 (48.9)
Igbo Eze South	5	63 (7.3)	65 (7.5)	128 (14.8)
Nsukka	8	50 (5.8)	34 (3.9)	84 (9.7)
Udenu	5	81 (9.3)	91(10.5)	172 (19.8)
Uzo Uwani	7	21(2.4)	9 (1.0)	30 (3.4)
Total	35	420(48.4)	448 (51.6)	868

### HIV seropositivity amongst participants from different LGAs

Out of 868 subjects that participated in the study, 176 (20.3%) were HIV seropositive. Of these, 97 (54.5%) were females and 79 (45. 5%) males. The difference between male and female seropositivity was however not statistically significant (P < 0.05). Local government disaggregation of data on HIV seropositivity in both male and female participants showed that the highest prevalence (26.7%) was recorded in Udenu LGA, while the least (13.1%) was recorded in Nsukka LGA. The highest prevalence in males (30.0%) was found in Igbo Eze South and the least (9.5%) in Uzo Uwani LGA, while for females, the highest prevalence (33.3%) was found in Uzo Uwani and the least (11.6%) in Nsukka LGA (Table 2).

**Table 2:** HIV seropositivity amongst participants in different LGA

Male Subjects			Female Subjects		
LGA	Number Examined	Number Positive (%)	Number Examined	Number Positive (%)	Total Number Positive/LGA (%)
Igbo Etiti	18	4 (22.2)	11	2 (18.2)	6 (20.7)
Igbo Eze North	187	28 (14.9)	238	48 (20.2)	76 (17.8)
Igbo Eze South	63	19 (30.0)	65	13 (20.0)	32 (25.0)
Nsukka	50	7 (14.0)	34	4 (11.6)	11 (13.1)
Udenu	81	19 (23.5)	91	27 (29.7)	46 (26.7)
Uzo Uwani	21	2 (9.5)	9	3(33.3)	5 (16.7)
Total	420	79 (18.8)	448	97 (21.7)	176 (20.3)

### Prevalence of pulmonary TB in different LGAs in the zone

Of the 868 subjects that participated in the study, 192 (22.1%) tested positive to Ziehl Neelsen staining technique with an overall male to female preponderance of approximately 3: 2. The male to female preponderance of sputum smear positivity was consistent in all the LGAs sampled except for Uzo Uwani LGA, where there was female to male ratio of 55.6% to 25.9%. Analysis of the result showed that there was a significant difference (P < 0.05) between the prevalence of sputum smear positivity in male and female subjects that participated in the study. In terms of case detection in LGAs, Uzo Uwani recorded the highest prevalence of sputum smear positivity (33.3%), while Igbo Etiti had the least (13.8%) as shown in Table 3.

**Table 3:** Occurrence of Pulmonary TB in different LGA

Male Subjects			Female Subjects		
LGA	Number Examined	Number Positive (%)	Number Examined	Number Positive (%)	Total Number Positive/LGA (%)
Igbo Etiti	18	3 (16.7)	11	1 (9.0)	4 (13.8)
Igbo Eze North	187	47 (25.1)	238	41 (17.2)	88 (20.7)
Igbo Eze South	63	20 (31.7)	65	8 (12.3)	28 (21.9)
Nsukka	50	14 (28.0)	34	7 (20.6)	21 (25.0)
Udenu	81	21 (25.9)	91	20 (21.9)	41 (23.8)
Uzo Uwani	21	5 (23.8)	9	5 (55.6)	10 (33.3)
Total	420	110(26.2)	448	82 (18.3)	192 (22.1)

### TB and HIV co-infection

**Figure 2:**
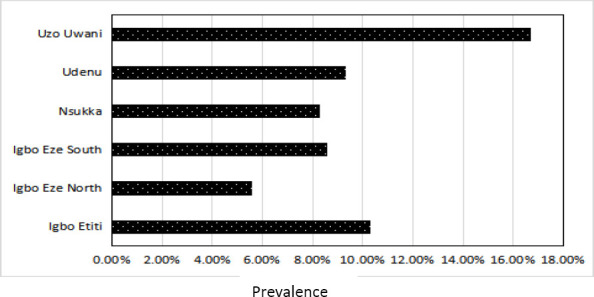
Prevalence of TB/HIV co-infection in different LGAs of Enugu North geographical zone

Among the 868 subjects included in this study, 66 (7.6%) had the double burden of HIV and TB. Local Government distribution of the co-infections of HIV/TB showed that Uzo Uwani LGA had the highest prevalence of 16.7%, followed by Igbo Etiti LGA (10.3%), while Igbo Eze North LGA had the least prevalence (5.6%), as shown in Figure 2.

### Report of TB prevalence by gender and age group

**Figure 3:**
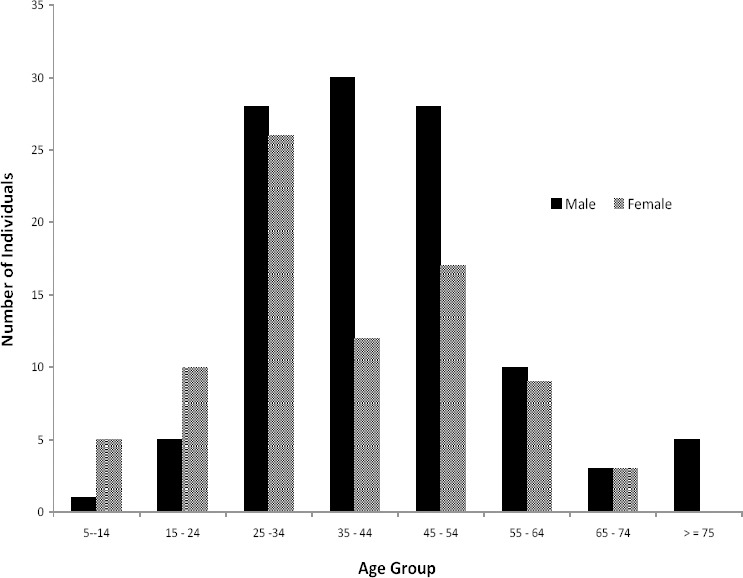
Distribution of TB positive cases amongst male and female participants in different age groups.

The highest prevalence of TB (34%) was found among male subjects in the age group of 35 – 44 years while that of female (27%) was in the age group 25 – 34 years. Amongst the young adults and middle aged, ranging from 25 to 64 years, sputum smear positivity was recorded more in male subjects within the different age brackets. However, amongst children and adolescents (5 – 24 years), results showed a female predominance of the sputum smear positivity (Figure 3).

### Prevalence of MDRTB in different LGAs

Of the 207 sputum samples subjected to NAAT (Xpert^®^ MTB/Rif test), 44 (21.3%) were positive. Of these, 6 (13.6%) were resistant. Nsukka LGA had the highest prevalence of resistant TB (30.0%), followed by Udenu LGA (16.7%), while Igbo Etiti and Uzo Uwani LGAs had the least prevalence with no resistance (Table 4).

**Table 4:** Prevalence of resistant TB in different LGAs

LGA	NAAT Positive Samples	Number Resistant to RIF	% RIF Resistance
Igbo Etiti	3	0	0
Igbo Eze North	12	1	8.3
Igbo Eze South	11	1	9.1
Nsukka	10	3	30.0
Udenu	6	1	16.7
Uzo Uwani	2	0	0
Total	44	6	13.6

## Discussion

The problem of TB globally has been found to be worsened by HIV/AIDS, as people with HIV have a much higher risk of developing active TB. According to health reports, about 45% of HIV-negative people with TB and nearly all HIV-positive people with TB, have a high risk of death, without appropriate treatment (WHO, 2020). HIV and TB, co-infection has been described as a lethal combination, as each disease speeds up the other’s progress; a realization which probably led to updating of the WHO TB/HIV treatment approach, in 2012, to place persons with HIV on TB-preventive therapy upon confirmation of HIV status, regardless of CD4 count (WHO, 2020). This makes it imperative that such cases be detected promptly and given appropriate treatment.

In this study, 868 subjects with cough, accessing treatment in DOTS centres within Enugu North geographical zone were screened for HIV seropositivity and TB. The prevalence of HIV among the subjects was 20.3%. The highest prevalence (26.7%) was recorded in Udenu LGA, while the least (13.1%) was in Nsukka LGA. Within the zone, Nsukka LGA represents the most cosmopolitan/urban area, within which a tertiary institution is located. The low prevalence of HIV in Nsukka LGA could be attributed to improved lifestyle and better enlightenment of the inhabitants. On the other hand, the high prevalence in Udenu LGA, though inconsistent with the reports of Okoli *et al.*, (2013), was not surprising, since the LGA houses one of the commercial nerve-centres of the zone, Obollo Afor, which is believed to have a high risk of HIV transmission due to increased number of commercial sex workers.

The overall prevalence of TB among participants was about 22% and there was a male to female preponderance of approximately 3:2,that was consistent in almost all the LGAs. This prevalence was higher than WHO projected national prevalence (WHO, 2018). Although the study was a hospital-based analysis (considering that most of the subjects were accessing chest clinics) and the findings may not be generalized in terms of prevalence of TB, the trends are nevertheless important. The higher male to female infection ratio, is in agreement with WHO reports, which state that globally, men are significantly more at risk of contracting and dying from TB than women. In 2017 alone, close to 6 million adult men contracted TB and around 840,000 died from it, compared to an estimated 3.2 million adult women who fell ill and almost half a million who died from the disease (Balasubramanian *et al.*, 2004; WHO 2018).

In this study, although Udenu LGA had the highest prevalence of HIV, the highest TB prevalence (33.3%) was recorded in Uzo Uwani LGA, despite contributing only 3. 4% of the total participants. In addition, the LGA also recorded the highest HIV/TB co-infection, suggesting a strong link between the TB infections and HIV, for this LGA. Moreover, this is the only LGA where there was a significantly (P<0.05) higher female to male ratio of infection, for both HIV and TB. This trend is potentially attributable to sociocultural factors. The LGA has a large population of women and widows, as there is a high death rate among the males. Furthermore, women in the area are known to frequently indulge in extra-marital affairs or prostitution for augmentation of income, after which they undergo a cleansing ritual, before returning to their husbands. This predisposes the women to high rates of HIV and TB infection. The overall prevalence of HIV/TB co-infection in this study was moderately high at 7.6%, while the prevalence in the different LGAs ranges from 5.6% to 16.7%. The findings are in line with previous studies conducted in Ethiopia (7.5%), Nigeria (7.8%) and Tanzania (8.5%) (Ngowi *et al.*, 2008; Iliyasu and Babashani, 2009: Wondimeneh *et al.*, 2012), but lower compared to 32.8% and 36.3% reported in some studies from Nigeria (Awoyemi, 2002; Egbe *et al.*, 2016). Analysis of TB prevalence, by gender and age-group also supports a strong link between TB and HIV in Enugu North geographical zone, as the TB cases were highest in the age groups, which are more active and have higher HIV risks, in both males and females. Age disaggregation showed that 34% of the overall sputum smear positive TB was found among male subjects aged 35-44 years while highest prevalence (27%) occurred in females aged 25 – 34 years.

In this study, the prevalence of drug-resistant TB was 13.6%. This finding is lower than the report of a study in southern Nigeria, with prevalence 23.4% (Adejumo *et al.*, 2018) and another from northern Nigeria with prevalence of 14.3% (Fadeyi *et al.*, 2017). However, the prevalence is relatively high in comparison with reports of studies from other parts of Nigeria and other African counties such as Ethiopia and Burundi (Sanders *et al.*, 2006; Osman *et al.*, 2012; Adane *et al.*, 2015; Egbe *et al.*, 2016; Kuyinu *et al.*, 2018). The variation in reported prevalence even within the same country may reflect the variations in sample size, access to health care facilities, and effectiveness of TB control programmes. A somewhat surprising finding from this study was Udenu LGA having the highest prevalence for HIV, while Uzo Uwani had the highest TB prevalence, and the highest prevalence of drug-resistant TB was in Nsukka LGA. This can, however, be attributed to the higher number of cases in male subjects, who are of course less likely to visit the hospital for care and more likely not to follow through with treatment, as suggested by other reports (Auld *et al.*, 2014: Bor *et al.*, 2015).

Non-completion of treatment has been the top factor among factors contributing to TB drug-resistance (WHO, 2020). It can be concluded from this study that Enugu North geographical zone, Nigeria, has a high prevalence of both HIV and TB, including multidrug resistant TB and there is need to increase monitoring of this region.

**List of Abbreviations:**
TB: – Tuberculosis
MDRTB: – Multidrug-Resistant Tuberculosis
WHO: – World Health Organization
HIV: – Human Immunodeficiency Virus
AIDS: – Acquired Immunodeficiency Syndrome
NTBLCP: – National Tuberculosis and Leprosy Control Programme
LGA: – Local Government Area
NPC: – National Population Commission
DOTS: – Directly Observed Treatment Short-course
NAAT: – Nucleic Acid Amplification Test

## References

[1] Adane K, Ameni G, Bekele S, Abebe M, Aseffa A (2015). Prevalence and drug resistance profile of mycobacterium tuberculosis isolated from pulmonary tuberculosis patients attending two public hospitals in East Gojjam zone, northwest Ethiopia. BMC Public Health.

[2] Adejumo O. A, Olusola-Faleye B, Adepoju V, Bowale A, Adesola S, Falana A, Owuna H, Otemuyiwa K, Oladega S, Adegboye O (2018). Prevalence of rifampicin resistant tuberculosis and associated factors among presumptive tuberculosis patients in a secondary referral hospital in Lagos Nigeria. African Health Sciences.

[3] Auld A, Shiraishi R, Mbofana F, Couto A, Fetogang E, El-Halabi S (2014). Lower levels of antiretroviral therapy enrollment among men with HIV compared with women - 12 countries, 2002–2013. MMWR:Morbidity Mortality Weekly Report.

[4] Awoyemi O. B, Ige M, Onadeko B. O (2002). Prevalence of active pulmonary tuberculosis in human immunodeficiency virus seropositive adult patients in University College Hospital, Ibadan, Nigeria. African Journal of Medicine and Medical Sciences.

[5] Balasubramanian R, Garg R, Santha T, Gopi P. G, Subramani R, Chandrasekaran V, Thomas A, Rajeswari R, Anandakrishnan S, Perumal M, Niruparani C, Sudha G, Jaggarajamma K, Frieden T. R, Narayanan P. R (2004). Gender disparities in tuberculosis:report from a rural DOTS programme in south India. International Journal of Tuberculosis and Lung Disease.

[6] Bor J, Rosen S, Chimbindi N, Haber N, Herbst K, Mutevedzi T (2015). Mass HIV treatment and sex disparities in life expectancy:demographic surveillance in rural South Africa. PLoS Medicine.

[7] Egbe K, Ike A. C, Aleruchi C (2016). Prevalence of tuberculosis and rifampicin resistance among patients seeking medical care in Nasarawa State north central Nigeria. Science Journal of Public Health.

[8] Fadeyi A, Desalu O. O, Ugwuoke C, Opanwa O. A, Nwabuisi C, Salami A. K (2017). Prevalence of rifampicin-resistant tuberculosis among patients previously treated for pulmonary tuberculosis in North-Western, Nigeria. Journal of the Nigerian Medical Association.

[9] Iliyasu Z, Babashani M (2009). Prevalence and predictors of TB co-infection among HIV-seropostitive patients attending Aminu Kano Teaching Hospital Northern Nigeria. Journal of Epidemiology.

[10] Kuyinu Y. A, Odugbemi B. A, Salisu-Olatunji S. O, Adepoju F. O, Odusanya O. O (2018). Characteristics of *Mycobacterium tuberculosis* positive patients screened for drug-resistant tuberculosis at a tertiary health facility in Lagos. Journal of the National Medical Association.

[11] (2012). National Population Commission.

[12] National Tuberculosis and Leprosy Control Programme (NTBLCP) (2017). Annual Review Meeting.

[13] National Tuberculosis and Leprosy Control Programme (NTBLCP) (2019). Annual Review Meeting.

[14] Ngowi B. J, Mfinanga S. G, Bruun J. N, Morkve O (2008). Pulmonary tuberculosis among people living with HIV/AIDS attending care and treatment in rural northern Tanzania. BMC Public Health.

[15] Okoli O. A, Ezekoye C. C, Ochiabuto O, Nwafor C. N, Ugwu S. U (2013). Detection of HIV-1 and -2 antibodies among selected secondary schools in Udenu L.G.A of Enugu State, South East, Nigeria. Open Journal of Medical Microbiology.

[16] Osman E, Daniel O, Ogiri S, Awe A, Obasanya O, Adebiyi E, Ige O, Oladimeji O, Dairo O. G, Declercq E, Gumusoboga M, Akang G, Bakare R. A (2012). Resistance of *Mycobacterium tuberculosis* to first and second line anti tuberculosis drugs in south west, Nigeria. Journal of Pulmonary and Respiratory Medicine.

[17] Sanders M, Van Deun A, Ntakirutimana D, Masabo J. P, Rukundo J, Rigouts L (2006). Rifampicin mono-resistant *Mycobacterium tuberculosis* in Bujumbura, Burundi:results of a drug resistance survey. International Journal of Tuberculosis and Lung Disease.

[18] Wondimeneh Y, Muluye D, Belyhun Y (2012). Prevalence of pulmonary tuberculosis and immunological profile of HIV co-infected patients in Northwest Ethiopia. BMC Research Notes.

[19] World Health Organization (2018). Guideline on management of drug resistance-TB. In WHO Treatment Guidelines for Drug-Resistant Tuberculosis.

[20] World Health Organization Nigeria (2019). WHO engages over 12,000 community informants fast-track efforts in finding 'missing TB cases'in Nigeria WHO Fact Sheet.

[21] World Health Organization (2020). Tuberculosis Fact Sheet.

